# Comparative Molecular Docking of Antitrypanosomal Natural Products into Multiple *Trypanosoma brucei* Drug Targets

**DOI:** 10.3390/molecules14041513

**Published:** 2009-04-14

**Authors:** Ifedayo V. Ogungbe, William N. Setzer

**Affiliations:** Department of Chemistry, University of Alabama in Huntsville, Huntsville, AL 35899, USA; E-mail: dayovictor2000@yahoo.com (I-V.O.)

**Keywords:** Docking, Antitrypanosomal agents, Natural products, *Trypanosoma brucei*, Protease, Sleeping sickness

## Abstract

Antitrypanosomal natural products with different structural motifs previously shown to have growth inhibitory activity against *Trypanosoma brucei* were docked into validated drug targets of the parasite, which include trypanothione reductase, rhodesain, farnesyl diphosphate synthase, and triosephosphate isomerase. The *in-silico* calculations predicted that lowest energy docked poses of a number of the compounds can interact with catalysis-dependent residues, thus making them possible catalytic inhibitors and of course physiologically active. Compounds that possess a number of hydrogen-bond-accepting and/or -donating groups like phenolics and quinones show extensive interactions with the targets. Compounds like cissampeloflavone, 3-geranylemodin and ningpogenin thus offer profound promise.

## 1. Introduction

Human African Trypanosomiasis (HAT) caused by *Trypanosoma brucei gambiense/rhodesiense* remains of one the world’s most neglected parasitic diseases, endangering millions of people and livestock in sub-Saharan Africa [1]. Although promising lead compounds to combat the disease have been discovered in the last decade, current treatments are still ineffective and have serious adverse effects. Thus, prospecting for antitrypanosomal agents is pertinent and requires continued effort. Compounds from nature or their synthetic homologues have, however, continued to provide a vast majority of drug leads in almost all disease conditions [2].

*In-silico* screening of small molecules has been in the forefront of drug discovery in recent years. Despite its array of applications in synthetic medicinal chemistry, its application to natural products drug discovery (NPDD) remains very sparse. There has, however, been much interest lately on the application of virtual screening to the highly laborious drug discovery efforts from nature and how it can stimulate the much needed renewed enthusiasm in NPDD [3].

A number of drug targets have been identified in *T. brucei*. These include trypanothione reductase (TR), rhodesain, triosephosphate isomerase (TIM) and farnesyl diphosphate synthase (FDS), in line with the fact that target-based drug discovery efforts remain a front runner in lead identification [4]. Coupled with this, several antitrypanosomal agents from plants have been characterized while considerable efforts are still being put into the search for more antiparasitic compounds that have been evolutionarily derived from nature, resulting from the co-existence of parasitic pathogens with other life forms. This work presents the molecular docking of antitrypanosomal natural products into *T. brucei* drug targets with a view of obtaining structural motifs that preferentially interact with these targets. Comparative docking of those compounds with the best binding properties to parasitic targets was also carried out with human homologues. This will not only help in designing synthetic analogues but also in planning extract purification and compound isolation schemes for target-based bioassays, which could lead to the isolation of potent antitrypanosomal agents that can become leads for drug development.

## 2. Results and Discussion

All the docked compounds have been previously reported to exhibit antitrypanosomal activity. The rerank score of the top poses calculated for all the compounds docked and their corresponding *in-vitro IC*_50_ against *T. brucei* cells in culture is presented in Table 1 below. The docking results revealed compounds with more favorable interactions with the targets and also indicated that some classes of compounds present certain structural motifs that could make them form extensive Van der Waals interactions and hydrogen bonding with targets.

### 2.1. Farnesyl Diphosphate Synthase

FDS catalyzes the formation of farnesyl diphosphate via consecutive condensation of two molecules of isopentenyl diphosphate with dimethylallyl diphosphate in the isoprenoid biosynthetic pathway, thus providing a precursor for the synthesis of ubiquinones, dolichols, sterols, heme *a*, and of course the prenylation of certain proteins [5]. Numerous reports have indicated that FDS is a therapeutic target in *T. brucei* [6,7]. Biphosphates, used in treating bone resorption, have been noted to have antiparasitic activity by way of FDS inhibition and are being investigated as potential antitrypanosomal drugs [8,9]. Table 2 below shows the top five compounds with favorable interactions with FDS. Angoroside C and cissampeloflavone led the top hit compounds for FDS based on the rerank score. Angoroside C has been previously shown to have moderate growth inhibitory activity against *T. brucei rhodesiense* [10]. Molecular docking calculations indicate that angoroside C and eleven other docked compounds (Table 1 and Table 2) have more favorable interactions with *T. brucei* FDS than the co-crystallized ligand, phenylalkyl bisphosphate-BPH-210, a potent inhibitor of FDS.

**Table 1:** Molecular docking pose scores of antitrypanosomal agents and the reported *IC*_50_ for *T. brucei*.

Compounds	Rerank Pose Score^a^				
	Rhodesain	Triosephosphate Isomerase (TIM)	Farnesyl diphosphate synthase (FDS)	Trypanothione reductase (TR)	*IC*_50_ for *T. brucei* (µM)
**Iridiods**					
6-*O*-methylcatalpol	-83.03	-86.80	-110.00	-104.04	86 [10]
6-*O*-β-d-xylopyranosylaucubin	-83.91	-77.47	-121.65	-83.30	79 [10]
Ajugol	-21.62	-87.10	-109.50	-102.89	91 [10]
Ajugoside	-78.81	-99.60	-113.83	-113.83	144 [10]
Aucubin	-80.26	-98.53	-101.28	-119.70	148 [10]
Catalpol	-66.34	-75.50	-75.14	-81.19	151 [10]
Ningpogenin	-101.68	-79.62	-124.27	-122.97	172 [10]
Scrolepidoside	-68.48	-68.83	-101.37	-89.86	49 [10]
**Isoquinone Alkaloids**					
Ancistroealaine A	-70.34	-68.84	-57.11	-74.17	8.25 [11]
Ancistrogriffine A	-67.72	-79.84	-77.90	-88.33	5.53 [12]
Ancistrogriffine C	-64.00	-86.71	-88.96	-78.06	7.85 [12]
Ancistrogriffithine A	-85.35	-95.68	137.47	-53.62	1.15 [12]
Ancistrolikokine D	-79.61	-81.58	-94.94	-68.74	6.93 [13]
Ancistrotanzanine A	-76.87	-90.86	-43.39	-53.78	1.7 [14]
Ancistrotanzanine B	-71.15	-89.98	-83.70	-73.43	1.7 [16]
Ancistrotanzanine C	-76.33	-84.97	-35.30	-6.62	3.2 [15]
Ancistrotectonine	-68.12	-84.82	-93.37	14.88	10.2 [15]
Aromoline	-29.94	-76.77	160.86	-51.33	1.48 [16]
Berbamine	20.95	-36.57	478.31	-24.28	2.6 [17]
Berberine	-82.07	-86.90	-92.33	-107.69	0.53 [17]
Dioncophylline E	-74.74	-93.87	-71.17	-88.05	2.1 [18]
Emetine	-69.79	-98.46	-111.37	-103.12	0.039 [17]
Korupensamine A	-69.39	-89.57	-76.20	-4.14	4.93 [19]
Nangustine	-29.03	-83.45	-88.71	-91.92	33 [20]
Pancracine	-64.51	-87.31	-86.07	-89.97	2.4 [20]
**Miscellaneous Alkaloid**					
3-*O*-Acetylsanguinine	-66.14	-77.68	-84.68	-85.43	3.5 [21]
**Miscellaneous Compounds**					
8-Hydroxyheptadeca-1-ene-4,6-diyn-3-yl acetate	-93.68	-109.03	-118.13	-132.24	0.46 [22]
8-Hydroxylheptadeca-4,6-diyn-3-yl acetate	-105.81	-100.58	-103.24	-127.20	18 [22]
16-Acetoxy-11-hydroxyoctadeca-17-ene-12,14-diynyl acetate	-88.38	-88.12	-94.64	-117.40	1.1 [22]
Aculeatin D	-58.63	-92.09	-122.28	-129.51	0.48 [23]
**Phenolics**					
1,7-bis(4-hydrophenyl)-heptane-3,5-dione	-42.99	-49.29	-76.29	-91.90	7.4-8.3 [24]
1,7-bis(4-hydrophenyl)-heptene-3,5-dione	-49.35	-53.42	-72.18	-97.44	8.4 [24]
Ambigol A	-83.14	-90.16	-96.41	-106.15	33 [25]^b^
Ambigol C	-85.42	-95.93	-101.70	-111.03	11 [25]^b^
Angoroside C	-63.53	-93.95	-152.16	-98.74	75 [10]
Chaetoxanthone A	-51.70	-52.59	-52.79	-43.16	12.69 [26]
Chaetoxanthone B	-47.56	-57.78	-48.26	-29.30	26.26 [26]
Cissampeloflavone	-105.24	-114.60	-141.43	-135.28	1.99 [27]
Letestuianin C	-73.82	-69.42	-89.89	-112.69	7.36 [24]
Piscatorin	-89.25	-110.52	-118.69	-123.04	6.10 [28]
Punicalagin	-31.50	10.55	389.14	189.60	1.75 [29]
Vismione D	-97.84	-102.02	-121.43	-127.88	22 [30]
**Quinoline Alkaloids**					
Cinchonidine	-40.27	-77.57	-93.47	-92.77	7.1 [17]
Cinchonine	-47.11	-80.25	-93.21	-114.67	1.2 [17]
Cryptolepine	-73.02	-82.82	-72.71	-90.88	0.6 [31]
Neocryptolepine	-73.12	-87.48	-78.55	-89.50	2.23 [31]
Quinidine	-61.61	-84.40	-84.17	-98.87	0.77 [17]
Quinine	-60.77	-84.20	-85.38	-114.96	4.9 [17]
**Quinones**					
2-(1-hydroxylethyl)naphtho[2,3-b]furan-4,9-quinone	-75.43	-90.81	-74.83	-99.66	0.05 [32]
3-Geranylemodin	-93.20	-97.03	-129.13	-138.76	35.4 [30]
4°-*O*-demethylknipholone-4°-*O*-β-D-glucopyranoside	-78.06	-92.32	-129.29	-115.47	1.2 [33]
Emodin	-79.37	-93.13	-82.46	-83.17	67 [30]
Gabroquinone A	-90.99	-96.68	-113.22	-70.45	11.3 [33]
Gabroquinone B	-91.59	-95.34	-114.86	-86.95	101 [33]
Isokigelinol	-64.21	-59.06	-68.31	-66.08	11.11 [32]
Isopinnatal	-55.03	-55.51	-77.04	-93.08	0.73 [32]
Kigelinol	-59.03	-74.05	-64.27	-70.88	21.28 [32]
Knipholone	-77.99	-100.24	-83.91	-63.32	21.4 [33]
**Terpenoids**					
Arnicolide A	-73.97	-73.66	-82.65	-56.31	1.42 [34]
Helenalin	-77.08	-71.00	-84.45	-63.25	0.051 [34]
Isoalantolactone	-61.91	-69.31	-62.40	-78.73	23.4 [34]
Ivalin	-56.83	-72.21	-66.62	-78.14	7.8 [34]
Mexicanin 1	-56.40	-62.47	-74.63	-76.12	0.318 [34]
Vernoguinoside	-86.81	-69.78	-108.12	-95.31	6 [35]
Vernoguinosterol	-60.89	-78.61	-107.89	-50.99	8 [35]

^a^ The rerank score is a linear combination of the E-inter (steric, Van der Waals, hydrogen bonding, electrostatic) between the ligand and the protein, and E-intra (torsion, *sp*^2^-*sp*^2^, hydrogen bonding, steric, Van der Waals, electrostatic) of the ligand weighted by pre-defined coefficients [49]. ^b^ Reported as MIC.

**Table 2:** Top hit compounds for docking to farnesyl diphosphate synthase (FDS).

Compounds	Class of Compound	Rerank Pose Score		H-bonding (kJ/mol)		E_Total _(kJ/mol)	
		*T. brucei*	Human	*T. brucei*	Human	*T. brucei*	Human
Angoroside C	phenolic	-152.16	80.14	-43.56	-33.83	-207.79	-56.03
Cissampeloflavone	phenolic	-141.43	54.45	-8.01	-15.44	-192.64	-88.88
4°-*O*-demethylknipholone-	quinone	-129.29	148.82	-41.50	-8.81	-159.31	-44.02
4°-*O*-β-D-glucopyranoside							
3-Geranylemodin	quinone	-129.13	-69.92	-13.43	-14.75	-147.73	-121.73
Ningpogenin	iridoid	-124.27	-79.16	-7.14	-10.41	-164.20	-94.61
BPH-210 ^a^	---	114.55	---	-8.65	---	-138.81	---

^a^ N-[methyl(4-phenylbutyl)]-3-aminopropyl-1-hydroxy-1,1-bisphosphonate; the co-crystallized ligand.

**Figure 1:**
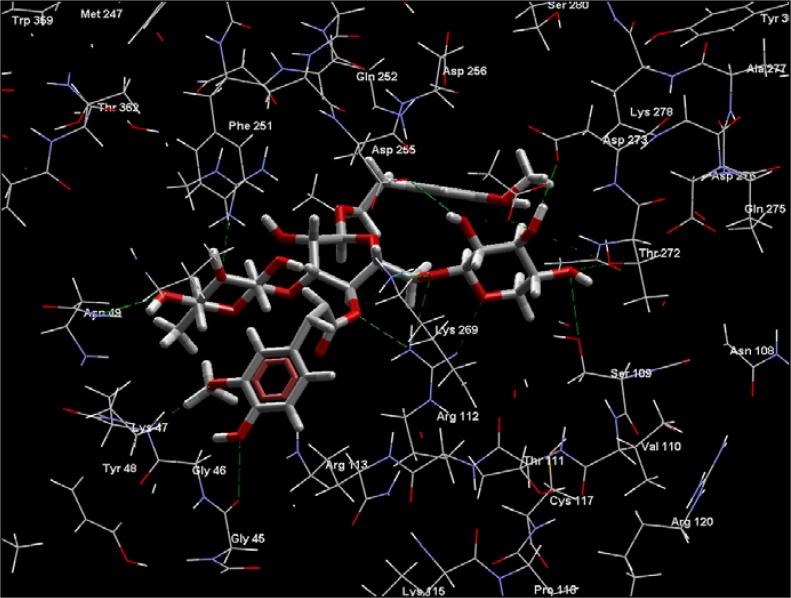
Some predicted interactions (green dashed lines) between angoroside C (white and red) and active site residues Arg 112, Ser 109, Lys 269, Gly 45, and Asp 255 of *T brucei* FDS.

**Figure 2:**
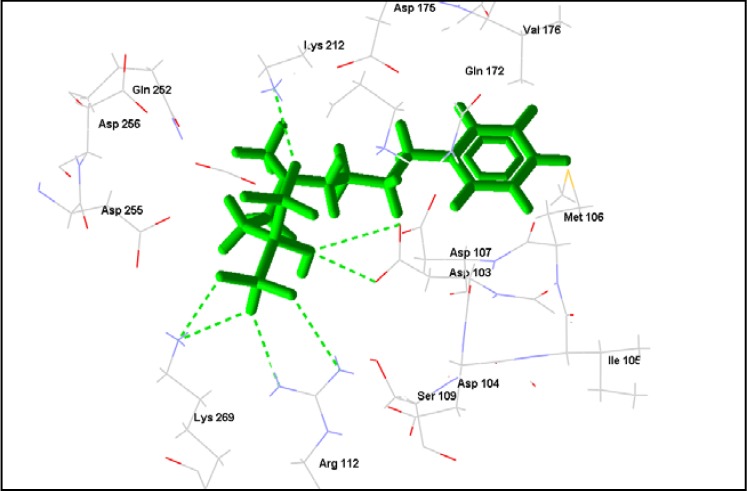
BPH-210 (green) in the active site of *T.brucei* FDS. Predicted hydrogen bonding interactions (green dashed lines) can be seen between BPH-210 and active residues Lys 212, Lys 269, Asp 103 and Arg 112.

**Figure 3:**
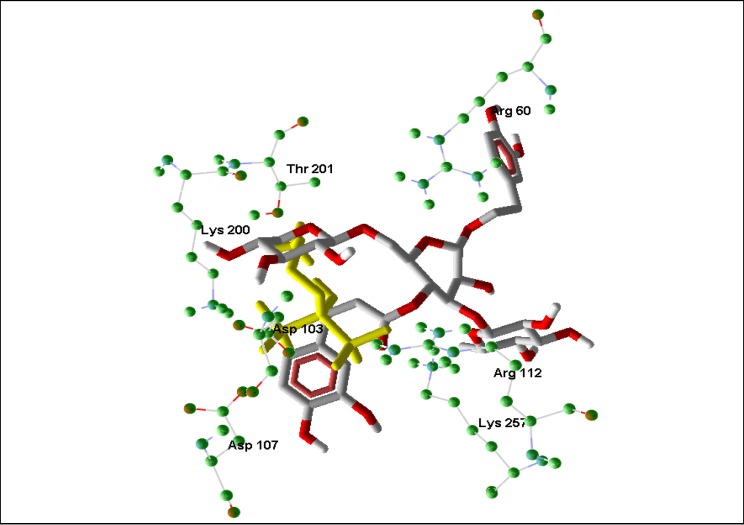
Top pose of angoroside C (white and red) and the co-crystallized ligand; alendronate (yellow) in the binding site of Human FDS, illustrating the orientation of important binding site residues Lys 257, Arg 112, Asp 107, Asp 103, Lys 200 around the phenolic compound.

Extensive interactions are observed between angoroside C and the Asp residues 103, 104, 107, 255, 259 of the protein’s repeating DDXXD motifs, Lys 212, 269, and Arg 112 (Figure 1); residues that have been noted as important for the binding and inhibitory action of BPH-210 (Figure 2) [36]. In comparison with the human FDS homologue, angoroside C and all the other compounds with top poses as shown in Table 2 have more favorable interaction energy based on both the rerank scores and total energies with the parasitic FDS than the human enzyme. Shown in Figure 3 is the orientation of angoroside in the isopentenyl pyrophosphate binding site of Human FDS.

Protein-ligand hydrogen bonding interactions are more extensive in the FDS-angoroside C complex compared to that of BPH-10, each having values -43.56 and -8.65 respectively (Table 2). Thus, phenolic and quinone compounds like angoroside C, cissampeloflavone and 3-geranylemodin could provide structural leads for more potent and selective FDS inhibitors.

Hydrogen bonding interactions between the ligand and protein are more extensive in the FDS-angoroside C complex compared to that of BPH-10 with a value of -43.56 to -8.6 respectively (Table 2). Thus, phenolic and quinone compounds like angoroside C, cissampeloflavone and 3-geranylemodin could provide structural leads for more potent and selective FDS inhibitors.

### 2.2. Rhodesain

**Figure 4:**
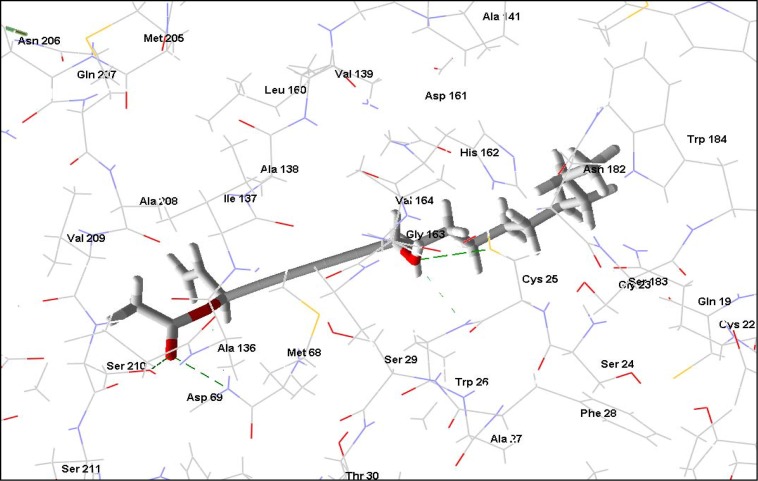
8-Hydroxylheptadeca-4,6-diyn-3-yl acetate interacting with rhodesain’s active site residues. Hydrogen bonding interactions are shown as green dashed lines.

**Figure 5:**
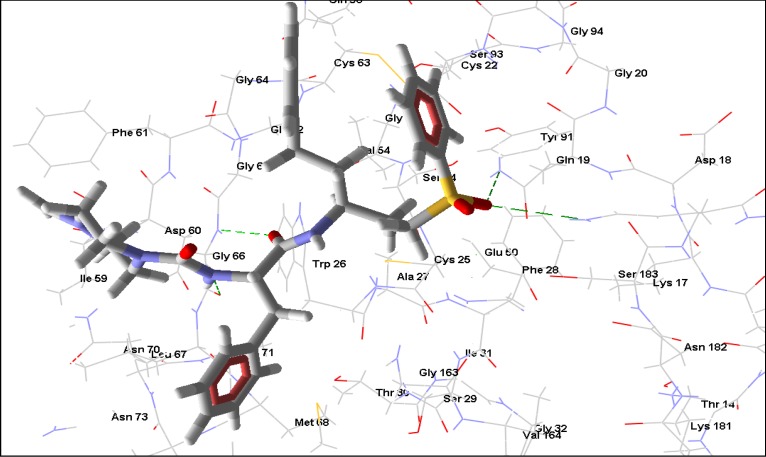
K777, rhodesain’s co-crystallized ligand, is shown interacting with the active site residues. Hydrogen bonding interactions are shown as green dashed lines.

**Supplementary Figure 1:**
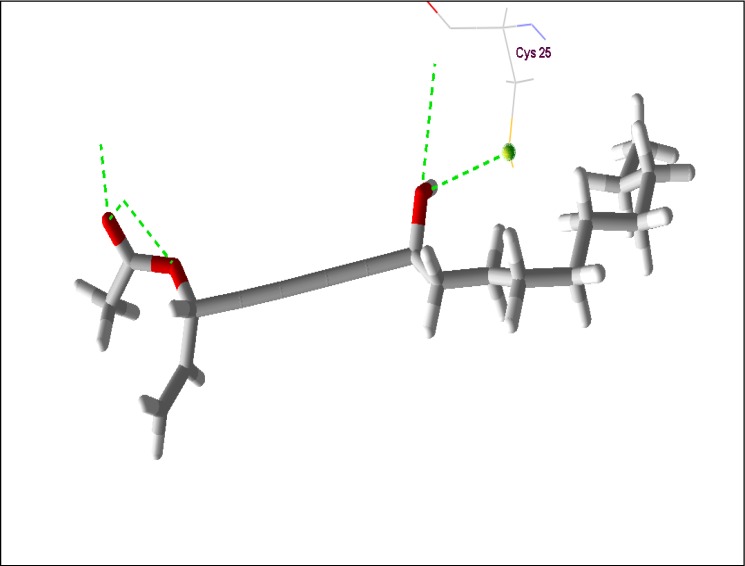
Predicted hydrogen bonding between 8-Hydroxylheptadeca-4,6-diyn-3-yl acetate and active site Cys 25.

**Supplementary Figure 2:**
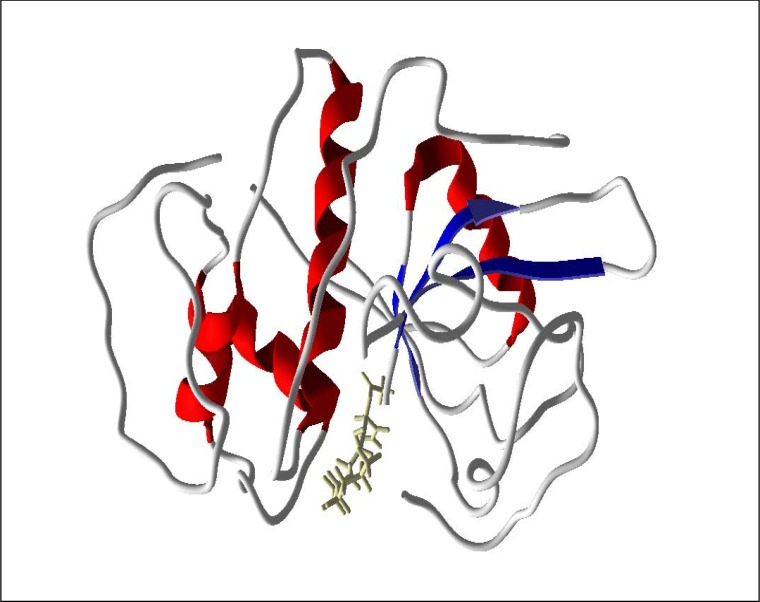
Orientation of 8-hydroxylheptadeca-4, 6-diyn-3-yl acetate along the cleft of rhodesain.

**Supplementary Figure 3:**
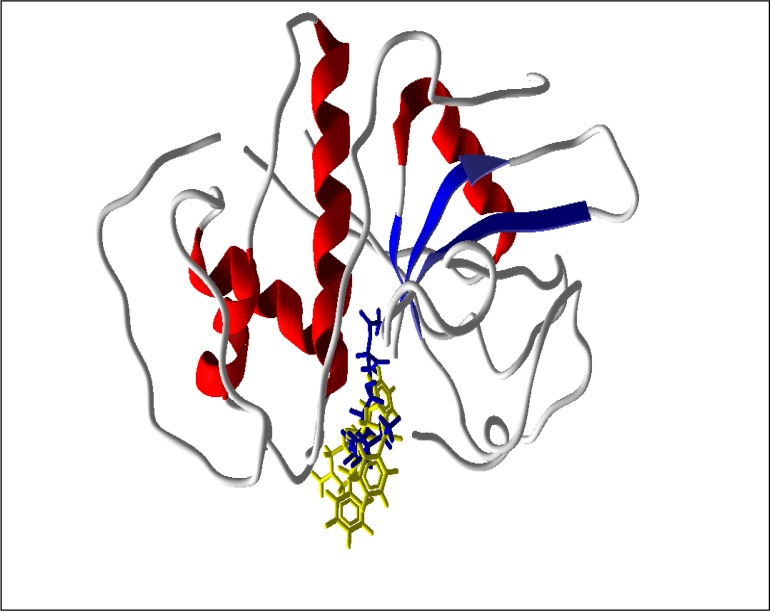
Superposition of 8-hydroxylheptadeca-4,6-diyn-3-yl acetate (Blue) on K777 (Yellow) along the cleft of rhodesain.

Parasitic proteases have been a subject of extensive investigations to identify novel antitrypanosomal agents due to the enormous roles they play in the parasites [37]. Rhodesain, the major cathepsin L-like protease in African trypanosomes, is a target for new HAT chemotherapy [38,39]. Interaction of compounds to the residues at the S4, S3, S2, S1 and the S1′, S2′, S3′ of the enzyme’s substrate binding cleft has been linked to their inhibitory activities [40]. Also, specific interactions with Cys 25, His 162, and Asn 182 that form the catalytic triad as well as Trp 184 and Gln 19 in rhodesain are essential. 8-Hydroxylheptadeca-4,6-diyn-3-yl acetate and 8-hydroxyheptadeca-1-ene-4,6-diyn-3-yl acetate, polyacetylenes from *Cussiona zimmermanni*, recently reported [22] to have antiparasitic activity, are among the five top compounds obtained from this docking study (Table 3). 8-Hydroxylheptadeca-4,6-diyn-3-yl acetate shows extensive interactions with residues along the substrate binding sites of rhodesain (Figure 4). Of particular importance is the predicted hydrogen bonding between the compound and the all important cysteine residue (Supplementary Figure 1) required for the protease’s activity. Target residues for K777 in the solved crystal structure and the docked complex of rhodesain and 8-hydroxylheptadeca-4,6-diyn-3-yl acetate indicate similar interactions with Ala 138, Asp 161, Cys 25, Gln 19, Gly 66, His 162, Leu 67, Trp 26, and 184 (Figure 4 and Figure 5, and Supplementary Figure 2 and Supplementary Figure 3). Cathepsin inhibitor selectivity remains an important issue when being considered for chemotherapy; this is not unconnected with the very similar specificity of cathepsins. However, structural differences are now being exploited in drug design. Poorer pose scores and weaker pose energies (Table 3) were obtained for the top compounds docked to the catalytic site of cathepsin L compare to the parasitic protease, indicating possible selectivity, with the interaction of 8-hydroxylheptadeca-4,6-diyn-3-yl acetate and the proteins revealing significant energy differences. Testing of this compound for inhibitory activity could provide valuable clue on its possible potency against the protease.

**Table 3:** Top hit compounds for docking torhodesain and corresponding energy values for human cathepsin L.

Compounds	Class of compound	Rerank Pose Score		H-bonding (kJ/mol)		E_Total_ (kJ/mol)	
		*T. brucei*	Human	*T. brucei*	Human	*T. brucei*	Human
8-Hydroxylheptadeca-4,6-diyn-3-yl acetate	diacetylene	-105.81	-92.08	-2.77	-2.70	-134.90	-109.49
Cissampeloflavone	phenolic	-105.24	-103.15	-4.81	-8.14	-159.29	-151.00
Ningpogenin	iridoid	-101.68	-66.52	-12.27	-8.05	-138.13	-81.82
Vismione D	phenolic	-97.84	-82.51	-2.50	-6.23	-110.79	-95.88
8-Hydroxyheptadeca-1-ene-4,6-diyn-3-yl acetate	diacetylene	-93.68	-92.38	-3.63	-2.78	-129.03	-113.74
K777^a^	---	-87.75	---	-6.61	---	-135.61	---

^a^ *N*-methylpiperazine-Phe-homoPhe-vinylsulfone-phenyl

### 2.3. Trypanothione reductase

**Figure 6:**
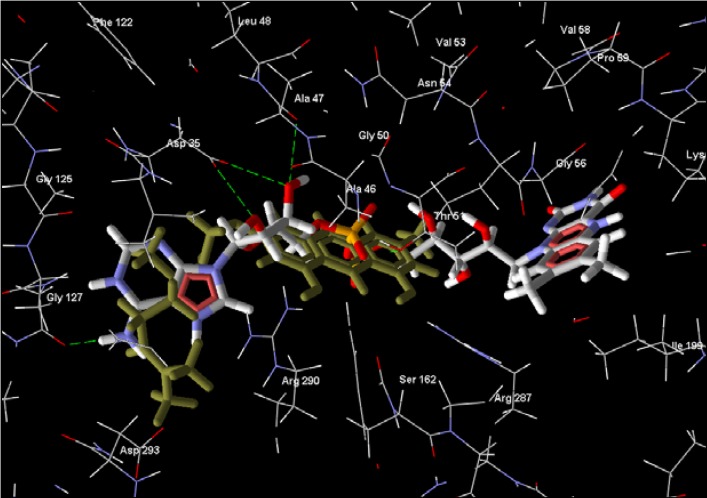
3-Geranylemodin (brown) and FAD (white, red and blue) interacting with residues at the FAD binding site of TR. Hydrogen bonding interactions are shown as green dashed lines.

**Figure 7:**
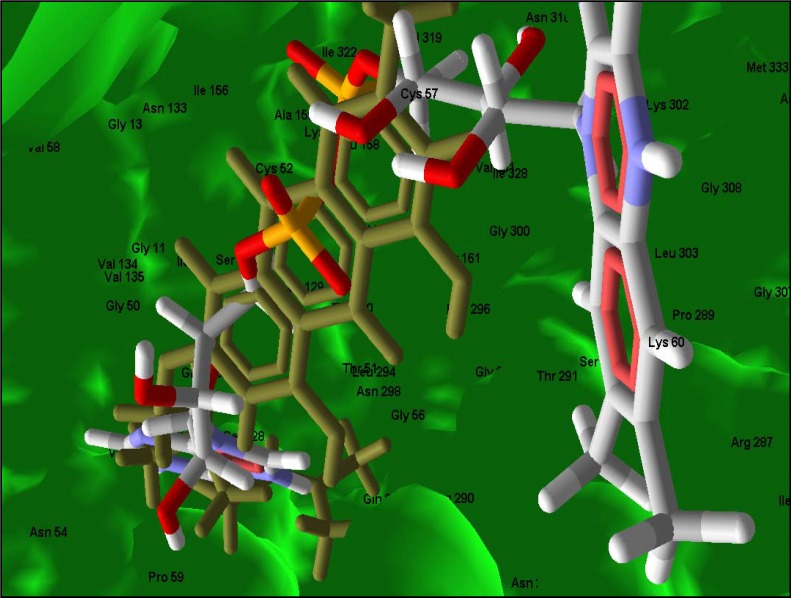
A close up on the orientation of 3-geranylemodin (brown) and FAD (white, red and blue) on a solid surface of TR.

TR is an attractive drug target in trypanosomatids due to its uniqueness in maintaining redox balance in these organisms, comparable to the ubiquitous glutathione/glutathione reductase system in mammalian hosts. Its vitality to the survival of the parasites has been reported [41]. The X-ray crystal structure of *T. brucei* recently solved by Jones *et al.* [42] provides a significant leap in pursuing *T. brucei* TR-selective inhibitors. Docking of these compounds into the predicted binding site of TR reveals that FAD has a comparatively higher rerank pose score and hydrogen bonding affinity than all the compounds (Table 4); however significant interactions are observed between the top pose of 3-geranylemodin and Ser 15, Gly 16 and Asp 329 residues of the protein. These residues have been previously noted to provide structural motifs for the binding of FAD (Figure 6 and Figure 7). In addition, predicted interaction of the ligand with Cys 53 at the disulfide substrate binding site, thus providing a long range interaction with the target residues, could be a major factor in its inhibitory potency. Of particular note is the fact that tricyclic compounds have been previously characterized as one of the known classes of TR inhibitors [4]. 3-Geranylemodin, a tricyclic anthraquinone previously shown to have moderate anti *T. brucei* activity is predicted from this calculation as the most likely TR inhibitor of the compounds considered. It also has a better ligand pose score for TR than for the human glutathione reductase, and this also occurs for the compound cissampeloflavone. In contrast, aculeatin D, vismione D and 8-hydroxyheptadeca-1-ene-4,6-diyn-3-yl acetate have poorer pose scores for TR but the total interaction energy between 8-hydroxyheptadeca-1-ene-4,6-diyn-3-yl acetate and TR is still more favorable than that of GR.

**Table 4:** Top hit compounds for docking to trypanothione reductase (TR) and corresponding values for human glutathione reductase.

Compounds	Class of compound	Rerank Pose Score		H-bonding (kJ/mol)		E_Total_ (kJ/mol)	
		*T. brucei*	Human	*T. brucei*	Human	*T. brucei*	Human
3-Geranylemodin	quinone	-138.76	-103.23	-15.08	-2.91	-207.63	-178.49
Cissampeloflavone	phenolic	-135.28	20.09	-5.62	-2.06	-159.57	-166.18
8-Hydroxyheptadeca-1-ene-4,6-diyn-3-yl acetate	diacetylene	-132.24	-155.71	-11.30	-2.72	-197.78	-181.85
Aculeatin D	miscellaneous	-129.51	-142.87	-1.14	3.38	-178.63	-182.96
Vismione D	phenolic	-127.88	-155.17	-6.95	-4.75	-155.87	-187.92
FAD^a^	---	-162.98	-295.81	-17.69	-33.86	-207.63	-351.32

^a^ Flavin adenine dinucleotide; co-crystallized ligand.

### 2.4. Triosephosphate Isomerase

**Figure 8:**
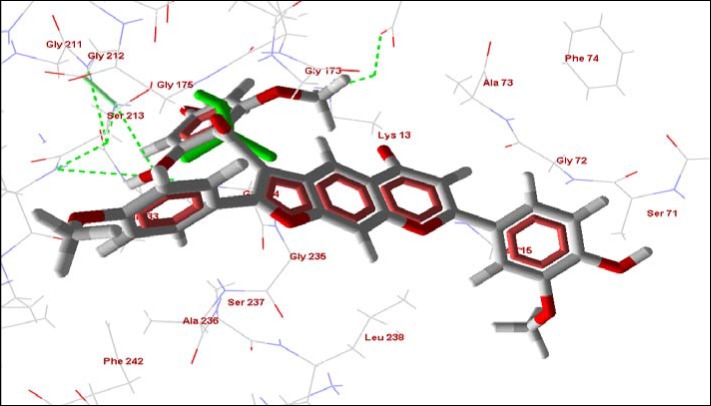
Cissampeloflavone (red and white) and HPO42- (green) at the phosphate binding site of TIM. Hydrogen bonding interactions are shown as green dashed lines.

**Figure 9:**
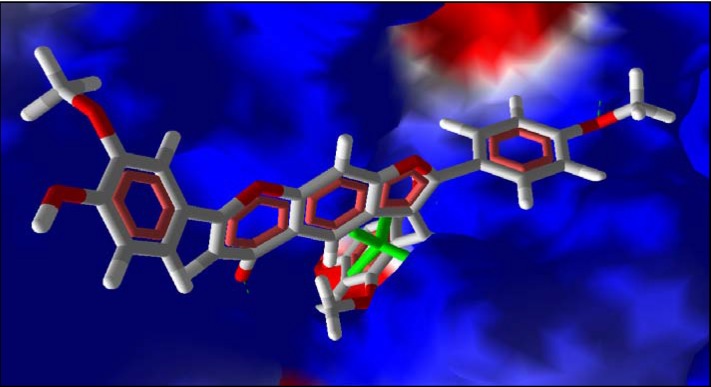
Electrostatic surface of TIM showing the insertion of a substituted phenyl ring of cissampeloflavone (red and white) into the HPO4^2-^ (green) binding groove.

TIM catalyzes the interconversion between glyceraldehyde 3-phosphate and dihydroxyacetone phosphate in the glycolytic pathway. The glycolytic pathway is particularly important in the bloodstream form of *T. brucei* due to their dependence on it for energy metabolism [43]. Although the ubiquitous nature of TIM and metabolic flux analysis have suggested that inhibiting glucose transport could prove more effective in killing trypanosomes by chemical agents [44], the structural differences in mammalian and trypanosomal TIM as reported by Olivares-Illana *et al.* [45] indicate, however, that *T. brucei* TIM remains a drug target and its specific inhibitors can be obtained from nature or designed. Table 5 below shows compounds predicted to have more favorable interaction with TIM, this enzyme possesses catalysis-dependent Glu-165 and His-95 and/or Lys-13 residues [46]. Each of these compounds is found to interact with these residues like the HPO_4_^2**-**^ group, a competitive inhibitor of TIM. One of the phenyl groups of cissampeloflavone is found to dock exactly at the HPO_4_^2**-**^ site with its methoxy and hydroxy substituents predicted to form extensive hydrogen bonding to nearby glycine and serine residues (Figure 8 and Figure 9). Considerably lower interaction energies are, however, obtained for the top poses docked to similar HPO_4_^2**-**^ binding site of the mammalian TIM (Table 5). These values give strong suggestions of significantly higher positive interaction between the ligands and the parasitic TIM in comparison to the mammalian TIM and of course this predicted selectivity can be exploited for drug design, synthesis and experimental validation.

**Table 5:** Top hit compounds for docking to triosephosphate isomerase (TIM).

Compounds	Class of compound	Rerank Pose Score		H-bonding (kJ/mol)		E_Total_ (kJ/mol)	
		*T. brucei*	Human	*T. brucei*	Human	*T. brucei*	Human
Cissampeloflavone	phenolic	-114.60	-9.91	-7.97	-2.50	-168.78	-21.80
Piscatorin	phenolic	-110.52	8.07	-6.70	-2.42	-144.90	-41.04
8-Hydroxyheptadeca-1-ene-4,6-diyn-3-yl acetate	diacetylene	-109.03	-34.68	-8.24	-1.10	-140.88	-52.57
Vismione D	phenolic	-102.02	-3.94	-9.78	0.00	-116.24	-10.10
8-Hydroxylheptadeca-4,6-diyn-3-yl acetate	diacetylene	-100.58	-38.15	-10.54	0.00	-132.00	-44.82
HPO_4_^2- ^	---	-38.82	---	-8.23	---	-47.00	---

### 2.5. Summary

The docking calculations in this study revealed that most of the compounds with top poses are phenolic and quinone compounds rather than the isoquinoline alkaloids out of the compound dataset used. Although these compounds have been shown to have growth inhibitory action against *T. brucei*, target-based screening of the compounds will provide information about selectivity and mechanism of action. It is important to note that since enzyme inhibition experimental data are not available for these compounds, structure-activity analysis thus becomes impossible, but target-based isolation schemes and/or synthesis of hit compounds predicted by *in-silico* investigations in conjunction with ethnomedicinal approaches is highly recommended and could eventually lead to new chemotherapy that can save humans and livestock from sleeping sickness in localities where the disease is endemic.

## 3. Experimental

### 3.1. Compound Dataset

**Figure 10:**
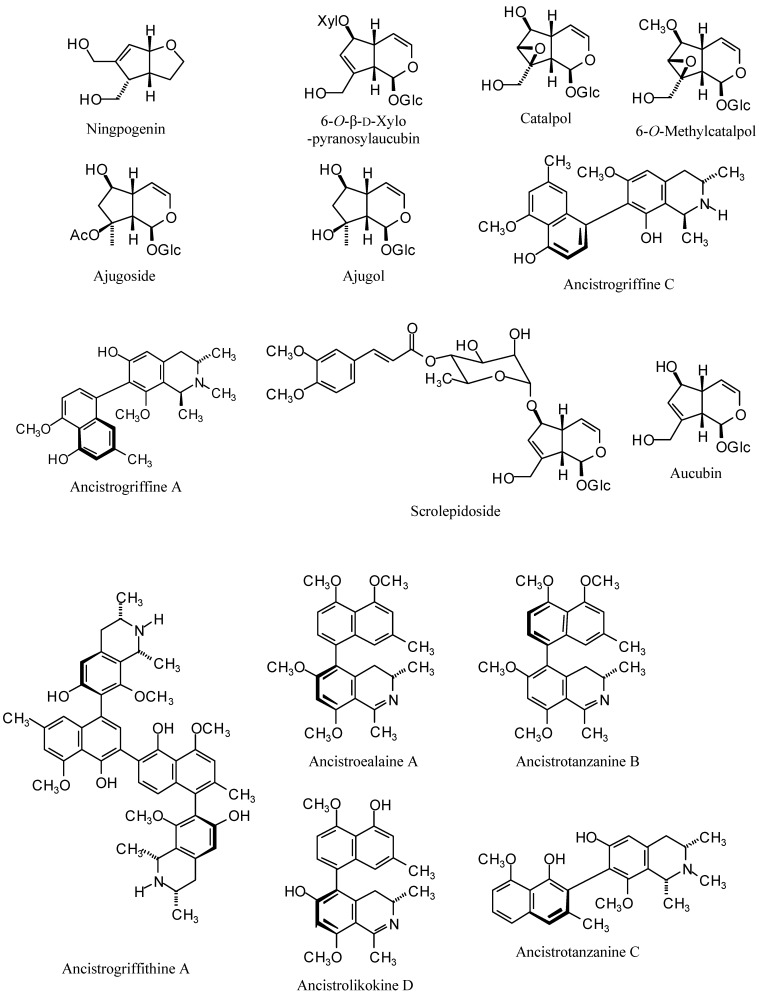

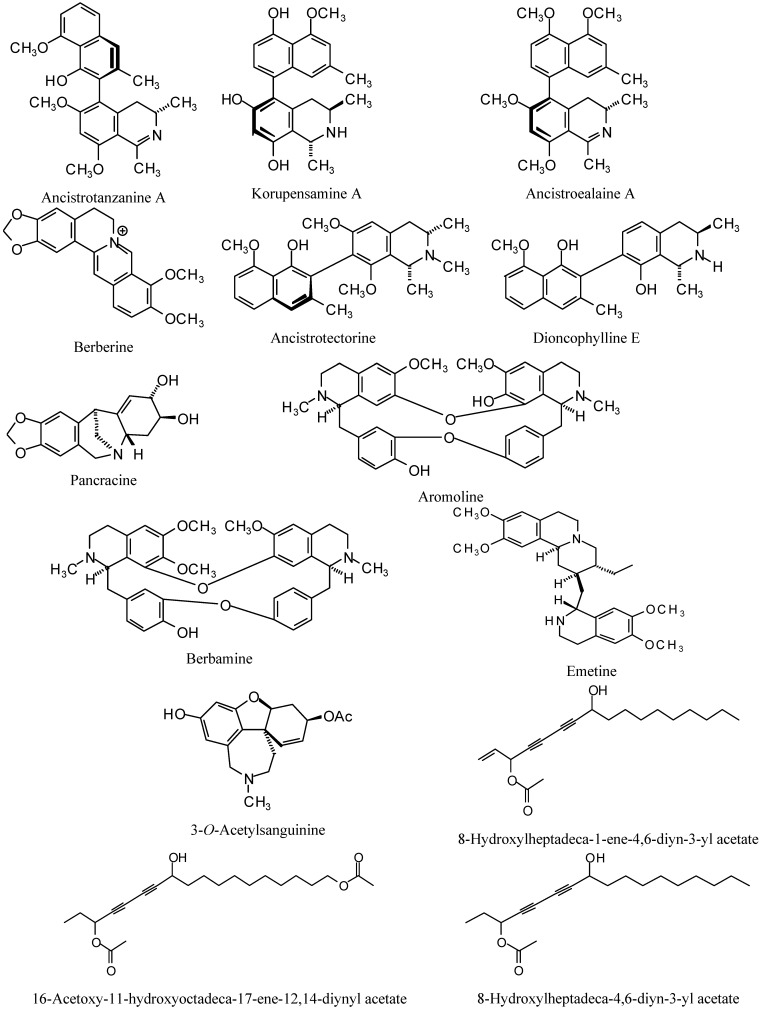

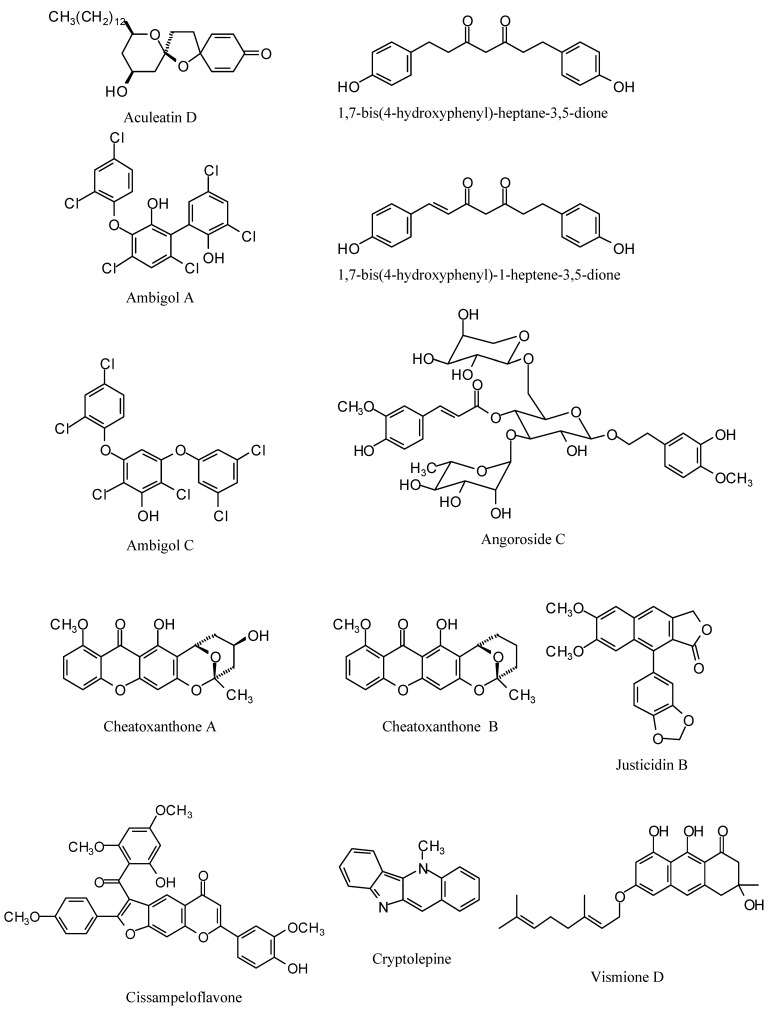

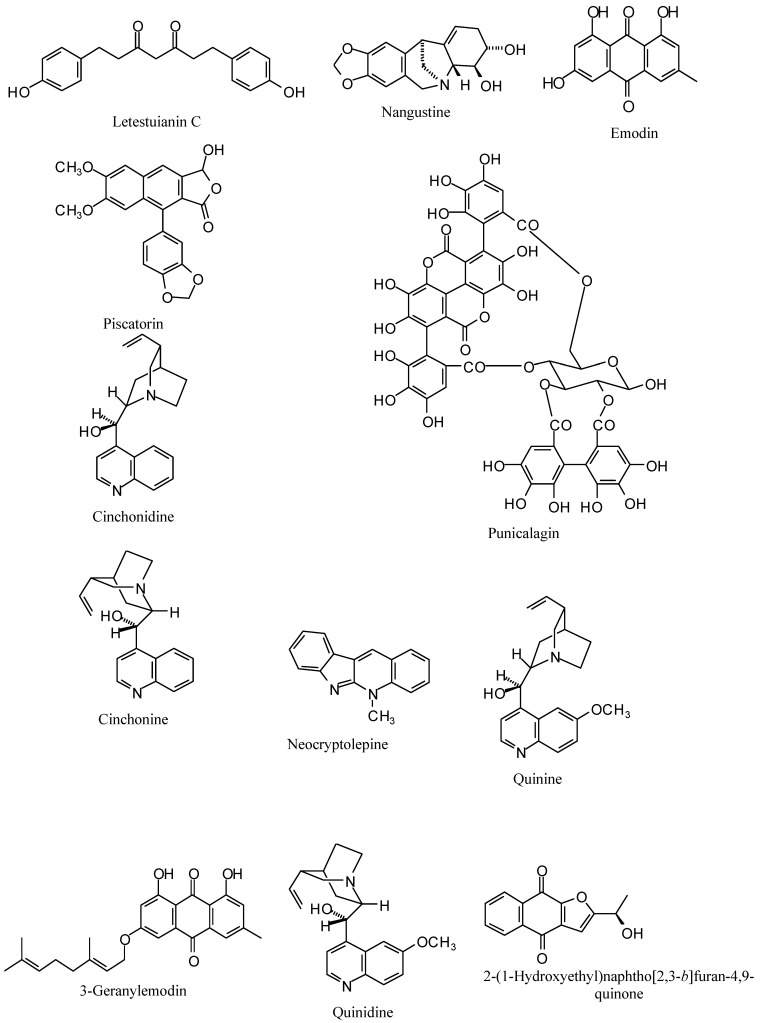

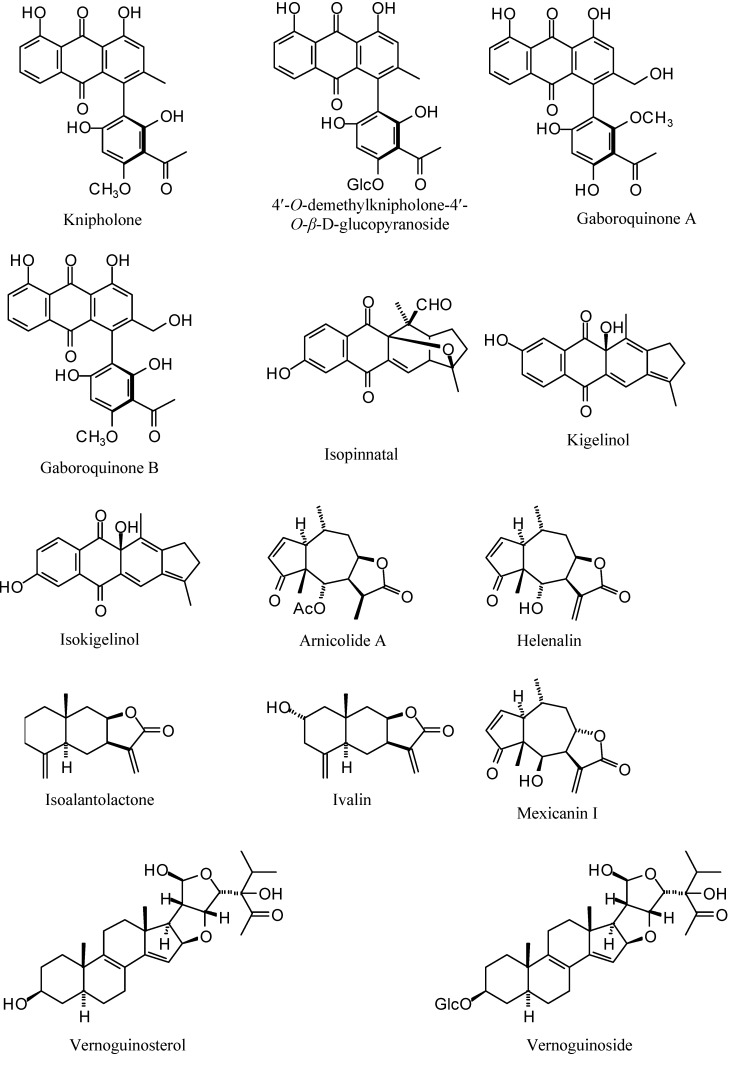
Structures of docked antitrypanosomal natural products discussed in this work.

Antitrypanosomal agents from plants (Figure 10) were obtained from the natural products literature. These compounds include iridoids, phenolics, terpenoids, alkaloids, quinones and a few miscellaneous compounds. Their exact structures were obtained from the Dictionary of Natural Products [47] and/or primary articles, drawn with correct stereochemistry using ChemSketch [48], energy minimized and saved as mol files.

### 3.2. Computation

**Supplementary Figure 5:**
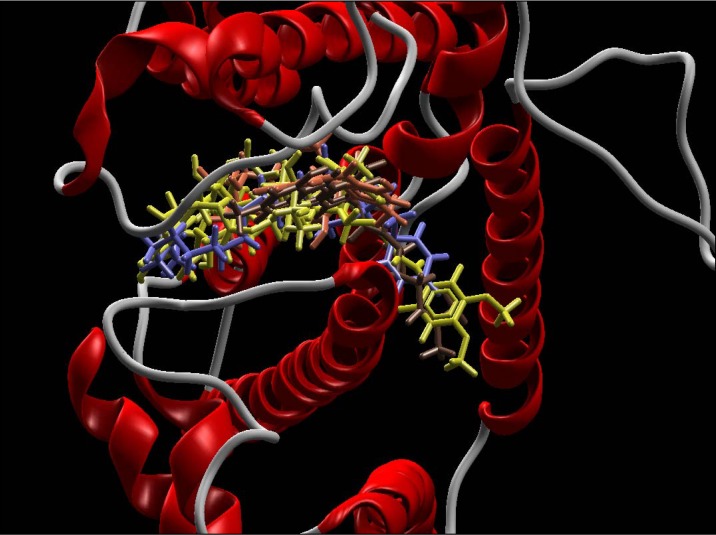
Superposition of the five compounds with top poses (shown with different colors) docked to FDS, indicating similar binding mode.

**Supplementary Figure 6:**
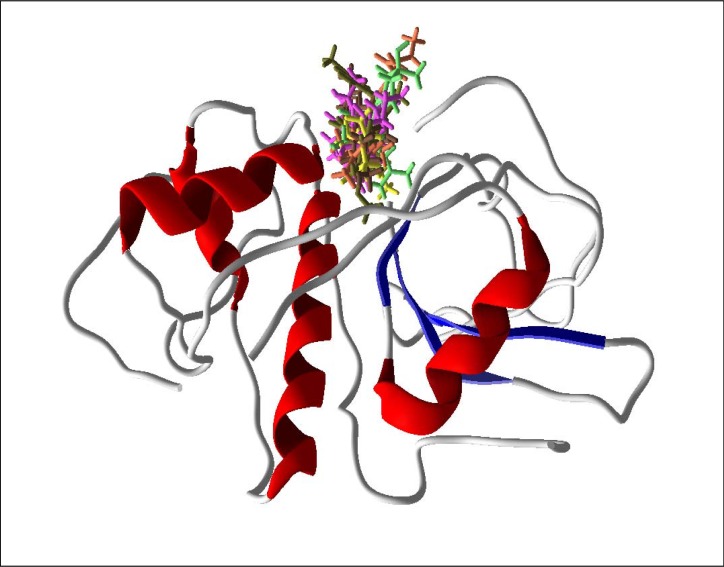
Superposition of the five compounds with top poses (shown with different colors) docked to rhodesain, indicating similar binding mode.

Molecular docking was carried out using Molegro Virtual Docker (MVD) [49]. MVD is based on a differential evolution algorithm; the solution of the algorithm takes into account the sum of the intermolecular interaction energy between the ligand and the protein, and the intramolecular interaction energy of the ligand. The docking energy scoring function is based on a modified piecewise linear potential (PLP) with new hydrogen bonding and electrostatic terms included. Full description of the algorithm and its reliability compared to other common docking algorithm can be found in reference [49]. The small molecules and the PDB crystal structure atomic coordinates determined by x-ray crystallography of TIM, rhodesain, FDS and TR [PDB Id: 1AG1, 2P7U, 2P1C and TVE2 respectively] were imported, potential binding sites were predicted. The binding cavity was set at X: 46.05, Y: 16.79, Z: -10.89 for TIM, X: 67.89, Y: 37.05, Z: -2.46 for FDS, X: 8.81, Y: 24.62, Z: 22.66 for TR and X: -8.23, Y: 2.30, Z: 10.28 for rhodesain. RMSD threshold for multiple cluster poses was set at < 1.00Å. The docking algorithm was set at maximum iterations of 1500 with a simplex evolution population size of 50 and a minimum of 10 runs. Representative superposition of poses of docked compounds is shown for FDS and rhodesain in Supplementary Figure 5 and Supplementary Figure 6 to show that docked poses have similar binding modes.

## Figures and Tables

**Supplementary Figure 4:**
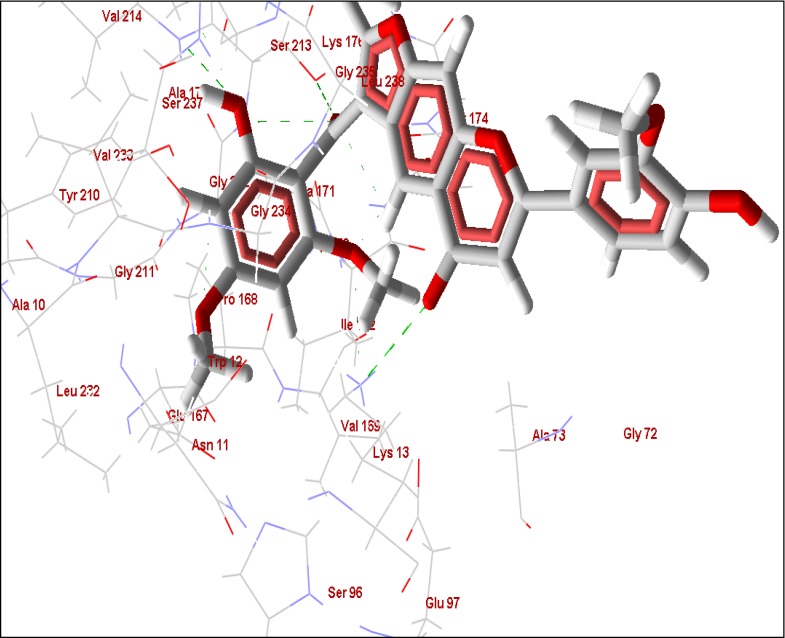
Predicted orientation of cissampeloflavone around the glycine and serine residues at the HPO_4_^2-^ binding site.
